# Evaluation of a Dutch school-based depression prevention program for youths in highrisk neighborhoods: study protocol of a two-armed randomized controlled trial

**DOI:** 10.1186/1471-2458-12-212

**Published:** 2012-03-20

**Authors:** Karlijn CM Kindt, Rinka van Zundert, Rutger CME Engels

**Affiliations:** 1 Behavioural Science Institute, Radboud University Nijmegen, Nijmegen, P.O. Box 9104, 6500 HE Nijmegen, The Netherlands; 2 Behavioural Science Institute, Montessorilaan 3, 6525 HR Nijmegen, The Netherlands

**Keywords:** Depression, Prevention, Adolescents, High-risk, School-based, Cognitive distortions, Alexithymia

## Abstract

**Background:**

Research has indicated that depression prevention programs attenuate the development of symptoms of depression in adolescents. To implement these programs on a large scale, implementation in a school setting with teachers providing the programs is needed. In the present study, the effectiveness of the Dutch depression prevention program Op Volle Kracht (OVK) provided by school teachers during school hours with adolescents from high risk neighborhoods will be tested. The mediating effects of cognitive distortions and alexithymia will be evaluated as well. We hypothesize that the OVK program will prevent or decrease reported depressive symptoms, and that this association will be mediated by cognitive distortions and alexithymia.

**Methods/Design:**

Schools with at least 30% of their pupils living in low income areas in the Netherlands are invited to participate in the study. Classes from vocational training up to pre-university level are eligible and 1324 adolescents (11-14 years) will be participating in the study. Randomisation will be done at class level, randomly assigning participants to an intervention group (OVK) and a control group (care as usual), stratifying by school level (high versus low). Trained school teachers will be delivering the program, which covers cognitive-behavioral and social problem-solving skills. Longitudinal data will be collected with self-report measurements administered in the school setting at baseline, post intervention and at two follow ups (at 6 and 12 months). Primary outcome is the level of depressive symptoms, and secondary outcomes include: cognitive errors, response style, attributional style, alexithymia, stressful life events, substance use, happiness, and school grades.

**Discussion:**

If the OVK program proves to be effective when it is provided by school teachers, a structural implementation of the program in the school curriculum will enhance the quality of the lives of adolescents and their families and will reduce costs in health care. In addition, the results of the study advances current knowledge on the underlying mechanisms of the development of depression and may aid the improvement of depression prevention programs in general.

**Trial registration:**

Dutch Trial Register NTR3110

## Background

Research pointing to the importance of cognitive distortions in the etiology and maintenance of depressive feelings has led to the development of cognitive behavioral interventions for youth. These interventions are not only used for the treatment of existing disorders, but also for the prevention of new cases. Recent meta-analyses suggest that preventive interventions can significantly reduce depressive symptoms or risk for future depressive symptoms compared to control groups [1-3]. More research is needed to test if depression prevention programs are effective when implemented in community settings under real-world conditions, and to uncover the causal mechanisms and potential mediators of the effects [1].

One of the best studied depression prevention programs is the Penn Resiliency Program (PRP) [4]. PRP is a theory-based universal school-based depression prevention program designed for early adolescents (ages 10-14 years) that teaches cognitive-behavioral and social problem-solving skills. Although PRP is described as not having replicated effects across trials in the meta-analytic review of Stice et al. [3], positive conclusions about this program are drawn in an evaluation of all of the 17 existing controlled studies evaluating PRP with 2,498 participants in total [1]. Researchers testing the Penn Resiliency Program often used rigorous experimental designs with extended follow-ups (up to 36 months) showing small but significant and consistent systematic effects on depressive symptoms, especially at 12-month follow-up assessments. Concluding, PRP seems to be a thoroughly studied theory-based program and a good basis for further development of depression prevention. In the Netherlands, no school-based depression prevention program for adolescents exists as of yet and therefore the PRP-program has been translated and rigorously adapted to the Dutch situation in close collaboration with the original developers. The Dutch program is titled "Op Volle Kracht" (OVK) [5]. The primary aim of the present study is to test the transportability and effectiveness of OVK when it is delivered by school teachers and provided to adolescents with a high risk to develop depressive symptoms (selective prevention).

Selective prevention implies focusing on a population whose risk is deemed to be above average to develop symptoms of a given disorder. This type of prevention has been found to be more effective than universal prevention [6,7]. Commonly distinguished groups with high risk for developing depressive symptoms are youth with: elevated depressive symptoms at baseline, a pessimistic explanatory style, parents with mood disorders, family conflicts, or youth who belong to an ethnic minority, live in low income areas and who are more exposed to life stressors [3,7-11]. Youth from families with a low social economic status are exposed to chronic levels of uncontrollable negative life events and to more maternal distress because of greater economic hardship [8]. Selective prevention is relevant for people in the teenage years [6], because depression rates begin to rise in early adolescence with a peak in mid-late adolescences [12]. In the present study, a selective prevention program for depression is targeted at adolescents living in low income areas in the Netherlands.

Since the purpose of the OVK program is to *prevent *youth from developing depression symptoms, a structural, easily accessible implementation on a large scale is needed to reach early adolescents who have not developed clinical depressive symptoms yet. This will be achieved by implementing the program in the regular school curriculum. No stigmatization will occur as a result of singling out individuals to receive the program. In addition, we expect the effects of the class-based program to be more easily consolidated because the classmates will interact and learn from each other in day-to-day experiences. For a feasible implementation of the program in the school curriculum, the following pragmatic and logistic arguments and costs have to be taken into account. Because hiring external professionals for providing the program is too expensive for most schools, it is important that school teachers are able to deliver the program. It is therefore pivotal to study if school teachers can provide the program as effectively as clinicians and/or the developers of the program, and to evaluate practical implementation challenges and difficulties. Meta-analyses of the PRP program show significant effect sizes regardless of group leader type [1], although it appears that effect sizes are higher when the program is delivered by professional interventionists compared to endogenous providers (e.g. teachers) [3,4,11].

### Mechanisms

In addition to studying the main effects of this program on symptoms of depression, the mechanisms and possible factors underlying the effects of the program are of great interest. To this end, the mediating effects of two specific concepts will be examined in the current study: distorted cognitions and alexithymia. Distorted cognitions are important determinants of depressive feelings. Three central theories explain the etiology and maintenance of depressive feelings with the role of cognitions: Beck's cognitive theory of depression [13], the hopelessness theory of depression [14] and the response styles theory [15]. According to Beck's theory, stressful events activate maladaptive self-schemata (i.e. a style of cognitive interpretation) which generates specific cognitive errors such as 'overgeneralization' and 'catastrophizing'. The hopelessness theory [14] states that an attributional style with negative outcome expectancy and expectations of helplessness about changing the likelihood of occurrence of these outcomes are causal for developing a (subtype of) depression. People with a negative attributional style have a tendency to attribute negative events to stable, global and internal factors which leads to hopelessness and consequently to symptoms of depression. Both theories describe a diathesis stress component [16]; the cognitive styles are only activated if they are accompanied by negative life events. The response styles theory [15] argues that the severity and duration of the symptoms of depression are determined by three styles in which individuals respond to their symptoms of depression: rumination (excessive thinking about the same topic), distraction and problem-solving. In research, the response style 'rumination' has been found to have moderating effects on the relation between life events and depressive symptoms; adolescents with a rumination response style are more likely to experience depressive symptoms when reporting more life events [17]. To study elevations in depressive symptoms as a consequence of cognitive distortions, longitudinal studies are needed so temporal sequences can be analyzed [18]. Research on cognitive distortions using longitudinal designs is however very limited [19]. We hypothesize that the OVK program will prospectively decrease the cognitive distortions of the adolescents and in turn will influence the reported depressive symptoms.

Another theoretical concept we expect to be highly relevant in relation to depression prevention programs is alexithymia. Alexithymia refers to difficulties in experiencing and verbalizing emotions and difficulties in emotional self-regulation [20]. An impaired emotion processing ability is suggested to lead to negative mood states and support for this hypothesis is found with cross-sectional data: children who score higher on alexithymia are found to ruminate and worry more about emotion-evoking situations compared to children who score lower on alexithymia [21]. Diminished alexithymia, in turn, is associated with a reduction of depressive symptoms, and although it is still unclear if cognitive behavioural therapy can reduce alexithymia, the first hopeful results are reported [22]. We hypothesize that the OVK program leads to less alexithymia and subsequent lower levels of depressive symptoms.

The first goal of the current study is to test if the OVK program prevents symptoms of depression when the program is delivered by teachers during school hours to a whole class of adolescents. The second goal is to study the mechanisms and possible factors underlying the effects of the program.

## Methods/Design

### Design

**Figure 1:**
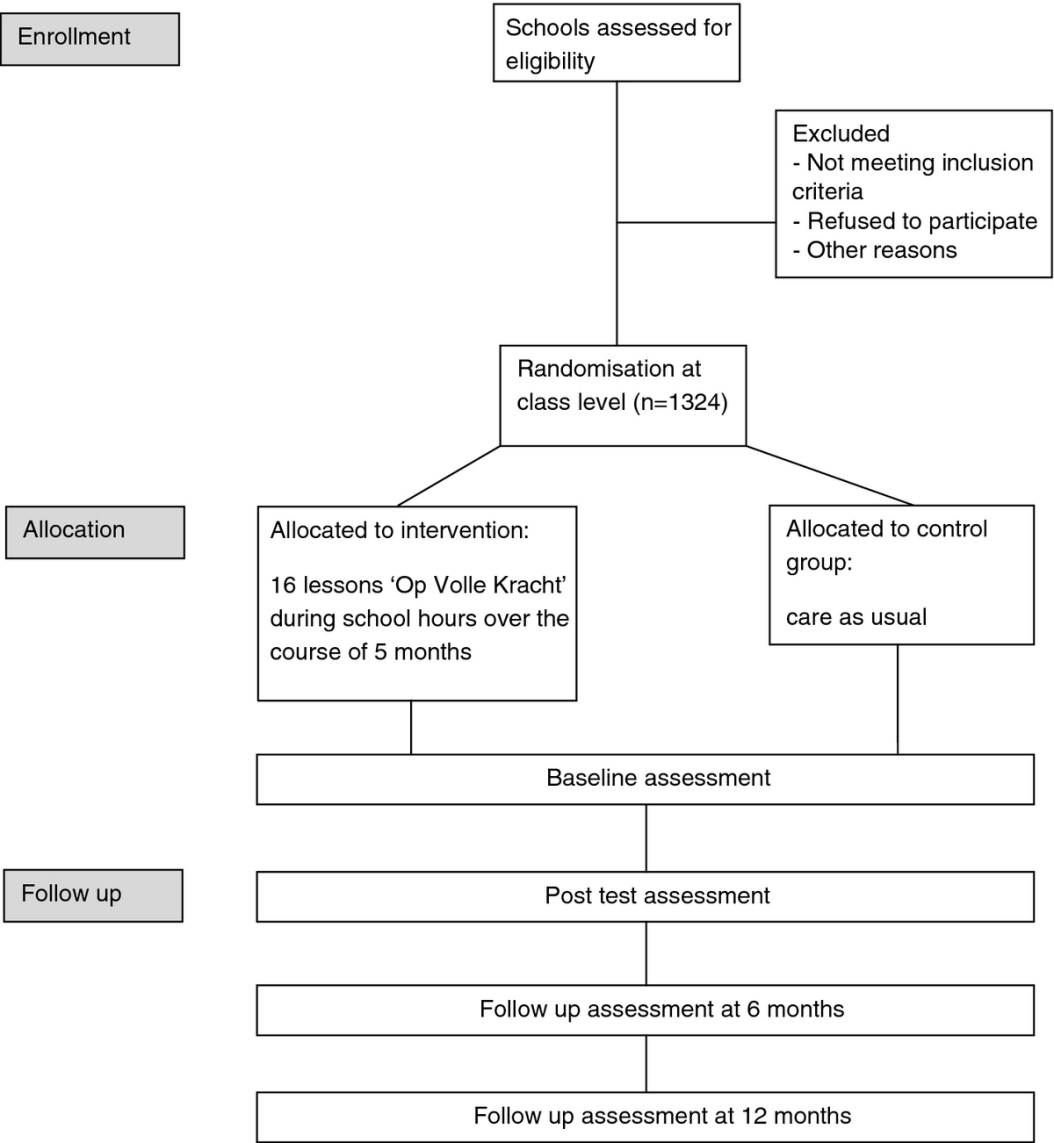
**Study design**.

The present longitudinal study involves a randomized controlled trial (RCT) with two conditions (intervention versus control) (see Figure 1) in which the effectiveness and the underlying mechanisms of the Dutch school-based depression prevention program Op Volle Kracht will be examined among adolescents from low income areas. The adolescents in the classes that will be assigned to the intervention condition will receive the 16-week OVK program from their teacher. Adolescents in the control condition will receive the usual school curriculum. While alternative interventions to control for nonspecific intervention ingredients are included in some of the PRP studies [4], the approach of 'care as usual' is chosen in the present study because no other school-based depression prevention program is currently implemented in the Netherlands. Still, some schools do provide social skill programs and we did not forbid schools to conduct those programs. The extent to which participants are involved in other prevention programs during the course of the study will be registered.

Assessments in both the intervention and control condition will be conducted through a 50-minute web-based self-report questionnaire during school hours that will be administered at baseline, post-intervention and at two follow-ups (at 6 and 12 months). Participants do not receive incentives for filling out questionnaires. A low attrition rate is expected, because the measurements are administered during the regular school hours. The teachers will be motivated to keep complying to the protocol through personal email contact with the research team in which they receive results about the assessments on school level.

### Participants and procedure

All the secondary schools in the Netherlands that meet the criterion of having at least 30% of their pupils inhabiting low income areas will be approached for participation in the study. In the Netherlands, this information can be obtained from the Dutch Ministry of Education, Culture and Science and it is based on the average income in the postal code area in which the pupils live. The eligible schools are predominantly situated in urban areas. Two other selection criteria are the educational level and age: pupils from vocational training up to pre-university level and from the 7^th ^and 8^th ^grade (range 11 to 14 years old) are selected. Two weeks after approaching the school by means of a written invitation, they will be contacted by telephone. If they are interested in participating in the study, a personal visitation will follow and classes will be selected. From the participating classes, none of the pupils will be excluded, to avoid stigmatization and social exclusion. With support from the schools, all parents will receive a letter to inform them about the content and aim of the program as well as the procedures of the study. Permission from the parents for their child's participation will be obtained through informed passive consent. If parents do not give permission for participation in the study, the adolescents will still attend the OVK program because it is taught in the regular school curriculum, but no data will be collected from them. To protect confidentiality of the data provided by the participating adolescents a unique code will be assigned to each participant. The Ethical Committee of the Faculty of Social Sciences at the Radboud University Nijmegen approved the study's protocol.

### Randomization

The classes will be randomly assigned to two conditions (intervention or control). An independent researcher will perform the allocation before the baseline assessment will be administered. Randomization will be carried out centrally on class-level and within schools to control for school characteristics, using a computerized random number generator with blocked randomization scheme (block size 2) and stratified by level of education (high versus low).

### Sample size

Power analysis (G-power) was conducted based on a 12-month effect size of .20. Although the expected effect size for a depression prevention program for high-risk youth is .40 (based on research of [11]; see also [3], the formulated effect size is more conservative, because the intervention will be conducted in classes of approximately 25 adolescents per class instead of 10 to 15 adolescents per group - as is done in some other school-based prevention programs [1]. For the power analysis, we considered potential loss of power due to clustering of data in groups at schools and due to multiple imputation and testing of mediational mechanisms. A maximum of 20% attrition over time has been taken into account as well. The sample sizes need to be 662 students per condition (alpha < .05, power = .80) and therefore the total sample size at baseline was determined to include 1,324 participants.

### Intervention

The OVK program will be taught by intensively trained school teachers and covers cognitive-behavioral and social problem-solving skills. Within 16 lessons of 50 minutes that are delivered during school hours, adolescents learn to detect inaccurate thoughts, to evaluate the accuracy of those thoughts, and to challenge them by considering alternative interpretations. They also learn techniques for assertiveness, negotiation, decision-making, social problem-solving and relaxation. The skills in the program can be applied to many contexts of life, including relationships with peers and family members as well as achievement in academics or other activities. Details about the content of the program are described extensively elsewhere [5]. To be able to deliver the program, the school teachers of the classes in the intervention condition will receive a 4-day training by two staff members of the research group; both of them are experienced and licensed psychologists and both are experts in cognitive behavioral therapy. The teachers receive a detailed manual of the program as guideline and they have the option to contact the trainers for additional support during program delivery.

### Assessments

The primary outcome is the level of symptoms of depression and will be measured with the Children's Depression Inventory (CDI) [23], which has been used in the vast majority of RCTs on universal depression prevention programs for youth and has good internal consistency and convergent validity [24]. The possibly mediating factors are cognitive errors, response style, attributional style, and alexithymia. The Children's Negative Cognitive Errors Questionnaire - Revised (CNCEQ-R) [25] will be used to measure five empirically derived negative cognitive error categories and is a revised version of the CNCEQ [26], which has a good internal consistency and test-retest reliability. The response style is measured with the Children's Response Styles Questionnaire [27] and consists of three subscales with moderate levels of internal consistency: ruminative response, distracting response and problem-solving. A shortened version of the Adolescent Cognitive Style Questionnaire [28] is used to measure attributional style. Adolescents rate the degree to which the cause of a hypothetical negative event is internal, stable and global. The scale has an excellent internal consistency and good test-retest reliability. Alexithymia is assessed with the subscales 'having difficulty identifying feelings' and 'having difficulty describing feelings' of the Toronto Alexithymia Scale (TAS20) [29,30] with good internal consistency and test-retest reliability.

Secondary outcomes are stressful life events, substance use, happiness, and school grades. The Adolescent Life Events Questionnaire - Revised [31] is used to assess the occurrence of a broad range of negative events which are typically reported by adolescents. The internal consistency of the scale is satisfactory. The Cantril Ladder will be used to measure happiness [32]. Academic performance will be assessed objectively by school grades we will receive from the schools. Socio-demographic variables of the adolescents and their family will be obtained by questions about sex, age, educational level, ethnicity, religious affiliation and psychiatric problems of the parents. The frequency and intensity of alcohol and tobacco use will be measured through commonly used questions that assess use of these substances [33-36].

### Statistical analysis

Data will be analyzed in accordance with the intent-to-treat principle but will also be analyzed separately for the completers only. Multiple imputations will be used for missing observations at follow-ups. The hypotheses will be tested with regression analyses for dichotomous and linear outcome measures in MPLUS 5.1 [37]. We will check for possible baseline differences between the two conditions in demographic variables (e.g. age, sex, school level, and ethnic background) and depressive symptoms. Moreover, variables that show different distributions between the two groups will be entered as confounders in all models testing the effectiveness of the intervention. The cluster effect - students are 'nested' in classes - will be handled by getting robust variance-related estimates using procedures for design-based analyses, (cf. [38]). We will correct for the cluster effects at class-level, as the interventions will be carried out in classes. Reporting of the results of the study will be in accordance with the CONSORT statement [39]. Mediation models will be tested with procedures as suggested by MacKinnon et al. [40].

## Discussion

The aim of the study is to test the effectiveness and mechanisms of change of a Dutch school-based universal depression prevention program 'Op Volle Kracht' for adolescents with high risk background. As we know very little yet about the mechanisms of change underlying effective CBT-based programs, we aim to test these mediating processes [19,41].

### Strengths and limitations

One of the strengths of this study concerns the rigorous research design of a randomised controlled trial with a large sample (N = 1,324) to analyse the underlying mechanisms and the sequence of change in the parameters. Another strength is the assessment of the cognitive distortions as possible mediators of symptoms of depression, which are based on three well-established cognitive theories [13-15]. Further, the added construct alexithymia has not yet been studied thoroughly in combination with depressive symptoms in youth. In addition, other highly relevant outcomes, such as academic achievement, will be measured to evaluate if other effects besides stimulating mental health might be reached by this program. Further, all assessments will be completed during school hours, which will ensure low attrition rates. An additional strength of the study is that the program will be delivered by teachers in a school setting, because the results will be of great importance in the discussion about practical implementation under real-world conditions (see also [1]). Last, the schools that will be participating in the study have a high diversity in school characteristics, e.g. Christian, Islamic, which generates a good external validity for the results of the study.

On the other hand, several limitations of this study exist. First, probably primarily motivated schools will join in the study, which may limit generalizability of the results to all schools. Second, by using a within-school design (as opposed to a between-schools design), contamination effects between the experimental and the control group could occur, although we expect to minimize these effects by delivering the program to entire classes instead of parts of classes. Third, the measurements are solely based on self-reports. Nevertheless, with respect to internalizing problems, it has been shown that adolescents are probably better informants compared to their parents or teachers [42]. At last, no integrity check of the delivery of the program will be conducted because of financial restrictions. However, the teachers will report on program adherence by filling out forms through which they report which (part of the) lessons they have covered.

### Implications for practice

If the OVK program proves to be effective in preventing elevated levels of depressive symptoms in adolescence, the study has strong practical relevance as the quality of life of the adolescents and their families will be enhanced and the costs for society and health care on the long term might be reduced. Because teachers are intensively trained, they are more likely to generalize their skills and knowledge to other parts of the curriculum. Moreover, they are expected to be more sensitive to signals of students with subclinical or clinical symptoms of depression and professional help could be consulted earlier. A structural implementation of the program in the school curriculum could be considered. In addition, the results of the study enable us to better understand the underlying mechanisms of development of depression and to improve depression prevention programs.

## Abbreviations

CNCEQ-R: Childrens Negative Cognitive Errors Questionnaire Revised; OVK: Op Volle Kracht; PRP: Penn Resiliency Program; RCT: Randomized Controlled Trial.

## Competing interests

The authors declare that they have no competing interests.

## Authors' contributions

All authors contributed to the design of this study. KK is responsible for the data collection and data analysis, as well as for reporting the study results. RvZ and RE are supervisors and principal investigators. All authors contributed to the writing of the manuscript. All authors have read and approved the final manuscript.

## Pre-publication history

The pre-publication history for this paper can be accessed here:

http://www.biomedcentral.com/1471-2458/12/212/prepub
